# Skeleton-Based Activity Recognition for Process-Based Quality Control of Concealed Work via Spatial–Temporal Graph Convolutional Networks

**DOI:** 10.3390/s24041220

**Published:** 2024-02-14

**Authors:** Lei Xiao, Xincong Yang, Tian Peng, Heng Li, Runhao Guo

**Affiliations:** 1 Department of Building and Real Estate, The Hong Kong Polytechnic University, Hong Kong, China; 1410213@tongji.edu.cn (L.X.); heng.li@polyu.edu.hk (H.L.); 2 School of Civil and Environmental Engineering, Harbin Institute of Technology, Shenzhen 518055, China; yangxincong@hit.edu.cn; 3 Technology Department, The Second Construction Company Ltd. of China Construction Second Bureau, Shenzhen 518000, China; pengtian@cscec.com

**Keywords:** activity recognition, construction, progress management, quality control, ST-GCN

## Abstract

Computer vision (CV)-based recognition approaches have accelerated the automation of safety and progress monitoring on construction sites. However, limited studies have explored its application in process-based quality control of construction works, especially for concealed work. In this study, a framework is developed to facilitate process-based quality control utilizing Spatial–Temporal Graph Convolutional Networks (ST-GCNs). To test this model experimentally, we used an on-site collected plastering work video dataset to recognize construction activities. An ST-GCN model was constructed to identify the four primary activities in plastering works, which attained 99.48% accuracy on the validation set. Then, the ST-GCN model was employed to recognize the activities of three extra videos, which represented a process with four activities in the correct order, a process without the activity of fiberglass mesh covering, and a process with four activities but in the wrong order, respectively. The results indicated that activity order could be clearly withdrawn from the activity recognition result of the model. Hence, it was convenient to judge whether key activities were missing or in the wrong order. This study has identified a promising framework that has the potential to the development of active, real-time, process-based quality control at construction sites.

## 1. Introduction

A certain type of construction work usually consists of several sequential activities, and the quality of work highly depends on the right order of activities. Subsequent activities may eventually cover the outcome of one activity, and the covered activity is called concealed work. It is difficult to assess in a non-destructive manner whether the concealed work has been carried out or not once the whole job has been completed. Thus, timely auditing of the process and results of the concealed work is vital to ensure that the work can be completed in steps, is compliant with procedures, and can be completed on time. Take the plastering work in decoration engineering as an example, which mainly contains four activities: surface preparation, fiberglass mesh covering, screeding, and coat application. The quality of each activity affects the quality of the follow-up activities. In particular, the fiberglass meshes can efficiently prevent cracks in the coat. However, the finishing coat will cover the fiberglass meshes after the whole process is completed, making it difficult to non-destructively detect whether the fiberglass meshes have been applied or not. Based on activity identification technologies, the order of activities can be identified and assessed automatically, which significantly facilitates progress management and quality control within the construction industry, especially for concealed work.

Due to the advancements in emerging technologies, automated real-time activity recognition systems for the construction industry have been developed in the last two decades [1]. Based on the types of sensors implemented, from both hardware and software perspectives, automated activity recognition methods could be divided into three major categories: kinematic-based methods, computer vision (CV)-based methods, and audio-based methods [2]. Kinematic-based methods use sensors such as accelerometers, gyroscopes, and inertial measurement units to recognize activities [3]. Audio-based methods mainly rely on recording sound patterns of equipment performing certain tasks [4,5]. CV-based methods identify activities by processing images or videos captured by different types of cameras [6]. Compared with the other two methods, CV-based methods are free of sensor–human contact and provide visual perception. Moreover, a large amount of visual data is affordable and easy to collect, since video surveillance is widely used for progress recording and site security.

Even though CV-based techniques are increasingly being used in construction, there is still significant untapped potential in this area. By far, CV-based techniques within the construction industry are mainly applied in safety [7,8,9,10,11,12,13] and progress monitoring [14,15,16]. Regarding construction quality control, most of these studies focus on surface quality evaluation, such as cracks, and few focus on the quality management of concealed work [17]. 

This study developed a CV-based framework that automatically identifies construction work activities and evaluates their orders. The framework is expected to be of particular value in process-based quality control, especially for concealed work. More specifically, a skeleton-based action recognition method, the Spatial–Temporal Graph Convolutional Network (ST-GCN), was innovatively employed for process-based quality control at construction sites. ST-GCN can effectively capture both spatial and temporal information by operating on skeleton joints along both space and time, and its efficiency and robustness in activity recognition have been proven [18]. ST-GCN and its variants have achieved many encouraging results [19,20,21] and have become one of the most frequently used methods for activity recognition [22]. 

In summary, the main contributions of this paper are as follows:

(1) Provide a practical activity-order evaluation framework based on ST-GCNs which can facilitate process-based construction quality control. The experimental results highlight the accuracy of the approach and the feasibility of the framework.
(2) Release a new plastering work video dataset containing both RGB and skeleton data to verify our model.

The rest of the paper is organized as follows. In Section 2, we introduce related works in the literature. Methodologies are described in Section 3. Section 4 presents the model to identify four primary activities in the plastering work. Section 5 covers an application example of the constructed model in order evaluation of the four activities, followed by discussions and conclusions in Section 6. 

## 2. Related Works

This section first introduces existing research works focusing on CV applications in construction. Then, the state-of-the-art methods for the considered problem, especially in skeleton-based human activity recognition, are reviewed.

### 2.1. Computer Vision in Construction

CV techniques can be grouped into three categories: object detection, object tracking, and action recognition [23]. These techniques have accelerated the automation of several construction management tasks that require extensive human involvement, including safety monitoring, progress monitoring, productivity analysis, and quality control [24].

CV plays a vital role in site safety management. It can help monitor unsafe conditions and actions continuously at a job site using visual data [25]. Seo et al. [23] derived a general framework for CV-based safety monitoring comprising three categories of approaches: scene-based, location-based, and action-based risk identification. Zhang et al. [26] further divided the application of CV techniques in workers’ health and safety supervision into two aspects, i.e., workers themselves and workers’ interactions with the site. Guo et al. [27] classified the application of CV in site safety management into three different levels: (1) detection, recognition, and tracking; (2) assessment; and (3) prediction. They suggested the second and third levels as future research directions. In light of advances made with deep learning, Fang et al. [28] reviewed its integration with CV to support behavior-based safety programs in construction.

Automated progress monitoring comprises three stages, i.e., (1) data acquisition, (2) as-built modeling, and (3) progress assessment [29]. Literature reviews in this domain have focused on at least one of the three stages. Omar et al. [30] presented a review of the acquisition of as-built data. Typical vision-based technologies used for site data collection include digital cameras, video cameras, laser scanners, and range imaging (or RGB-D cameras) [29]. Ma et al. [31] comprehensively studied techniques to generate as-built models. According to the algorithm and method employed, the as-built models can be classified into point-cloud models, voxel models, mesh models, surface models, and BIM models [29]. Progress assessment compares the as-built and as-planned models [32]. By far, three methods have been established in comparison with the as-planned status, including point cloud comparison, feature detection, and surface matching [33]. 

Productivity is defined as the ratio of output (e.g., the soil amount excavated and transported) to input resources (e.g., equipment hours on the worksites) [34]. Productivity analysis measures construction input and output, while progress monitoring focuses on construction output. Most of the literature on automated productivity analysis dives particularly into productivity analysis of heavy equipment. Kim et al. [35] combined vision-based context reasoning and construction process simulation for productivity analysis of an earthmoving activity. Chen et al. [36] proposed a framework that automatically recognizes activities and analyzes the productivity of multiple excavators. 

The use of CV in quality control is quite limited compared with its application in safety management and progress monitoring. By far, most studies have focused on surface quality assessment [37,38,39,40], but process-based quality control is understudied [41]. Luo et al. [17] provided a comprehensive literature review on digital technology for construction management from the perspective of the quality inspection process. They concluded that little attention was paid to the quality control of concealed works apart from surface quality assessment and measurements on concrete and steel. Quality control of concealed works in early construction projects was achieved mainly through enhanced collaboration and communication between project participants. Construction contractors submit applications to the quality inspection department for quality review and approval by the relevant personnel before concealed work commencement [42], which requires complex manual records and is very labor-intensive. Although Zhong et al. [43] proposed a method of ensuring the quality of construction by completing task lists, it is still difficult to ensure compliance with workers’ work behavior. Therefore, the existing research lacks a convenient and quick method for monitoring the quality of concealed works. This paper proposed a novel framework utilizing skeleton-based activity recognition, which has the potential to achieve a standardized inspection of the construction process and, thus, facilitate quality control of concealed works.

### 2.2. Skeleton-Based Human Activity Recognition

Human activity recognition based on human skeleton sequences has been extensively explored in recent years. Researchers usually represent the skeleton data with 2D or 3D human joint coordinates of all frames. Early studies on human skeletal action recognition methods generally focused on hand-crafted features [44]. 

Due to the abundant data and increased computing power, deep learning has become increasingly popular in the past few years. Deep learning has facilitated skeleton-based human action recognition by leveraging different aspects of skeleton data [44]. More specifically, recurrent neural network (RNN)-based methods enhance the order of joint coordinates by treating input skeleton data as time series [45]. Convolutional neural network (CNN)-based methods utilize spatial information by mapping skeleton sequences into pseudo-images representing temporal dynamics and skeleton joints in rows and columns, respectively [46]. Graph neural network (GNN)-based methods make use of both spatial and temporal data by exploiting information contained in the natural topological graph structure of the human skeleton [18]. GNN-based methods, in particular graph convolutional networks (GCNs), have become the most widespread method of performing skeleton-based action recognition. Models making use of GCN were first introduced in skeleton-based action recognition by Yan et al. [18], and they are usually referred to as Spatial–Temporal Graph Convolutional Networks (ST-GCNs).

In terms of skeleton-based activity recognition in construction, previous studies have mainly focused on identifying workers’ unsafe or non-ergonomic behavior [47]. Based on extracted human postures and pre-defined rules, Ray and Teizer [48] presented a real-time system that could determine whether a worker had the correct posture while executing characteristic construction work tasks. Guo et al. [49] use skeleton-based image technology to improve construction safety management by reducing dynamic movements to static poses in order to identify and warn workers of unsafe behavior. To the best of the authors’ knowledge, the application of skeleton-based activity recognition in construction quality control has not been reported. Moreover, the application of skeleton-based activity recognition can now benefit from the rapid advancement of image processing and skeleton extraction algorithms.

## 3. Methodology

### 3.1. Framework Overview 

**Figure 1:**
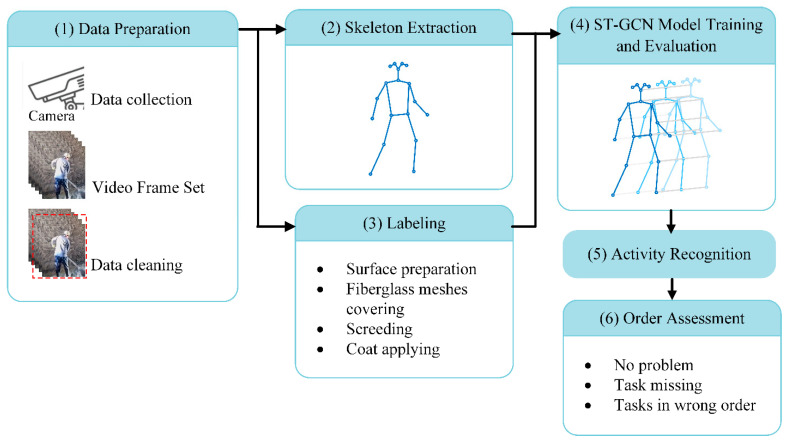
Pipeline of the presented framework.

To further explore the application of CV-based activity recognition techniques in process-based quality control in the construction industry, a methodology framework is proposed in this study. The method has the potential to automatically identify activities in particular construction works and assess their orders based on human skeletons without the need for previous knowledge of these activities. As shown in Figure 1, the real-time activity recognition and order assessment framework mainly consists of two algorithms for skeleton extraction and activity recognition, respectively. The video data collected from surveillance cameras are pre-processed before being put into the skeleton extraction algorithm to estimate the skeleton in every video frame. The extracted skeletons and labels of video clips are then used to train an ST-GCN model for action classification. Then, with each category of actions identified, the order of activities in an action sequence can be assessed automatically. Each procedure involved in the process shown in Figure 1 is described in detail in the following subsections. Specifically, this framework can be applied to different video scenes, regardless of the image background, the angle, and the distance of the camera.

### 3.2. Dataset Preparation

There are four steps to prepare the dataset, i.e., video collection, video segmentation, data cleaning, and annotation, which are described as follows:

(1) Video collection: To feasibly automatically recognize activities of particular construction works in real scenarios with the proposed method, the video dataset was collected based on surveillance videos from real construction sites.
(2) Video segmentation: Video segmentation is the process of slicing a continuous video into discrete portions for feature extraction. The videos were first converted to a frame rate of 30 frames per second (FPS), and then divided into segments of consecutive frames according to the categories of activities to build a dataset for each activity.
(3) Data cleaning: Invalid frames where the body or the operation of the worker was not fully captured by the camera or where irrelevant people were captured were removed.
(4) Annotation: The activity class labels were assigned to video segments according to the categories of activities. This step was intended to ensure that the skeletons over a period would correctly represent actual construction activities. In addition, this serves as the ground truth for the learning algorithm [3].

### 3.3. Skeleton Extraction

**Figure 2:**
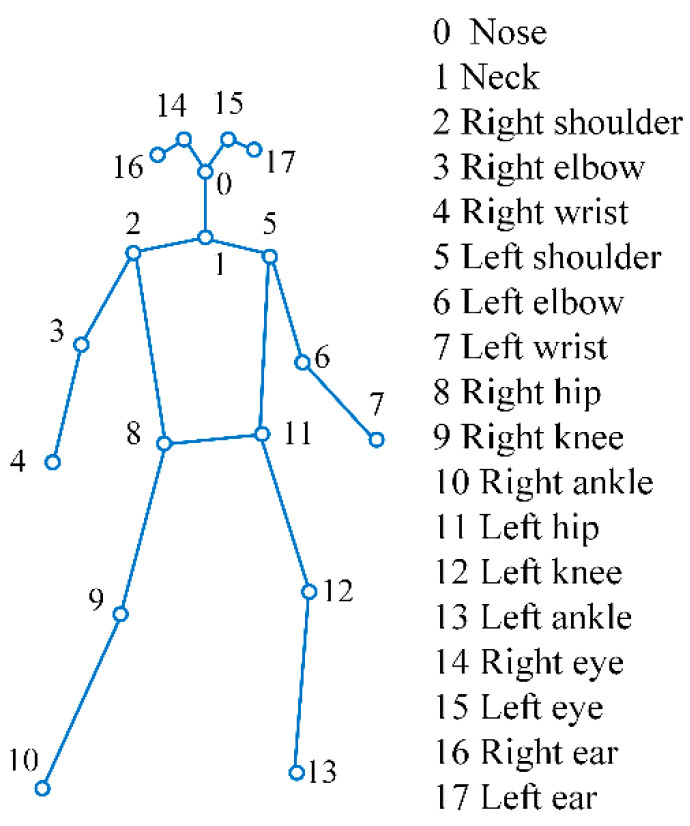
Skeleton joints provided by OpenPose.

**Figure 3:**
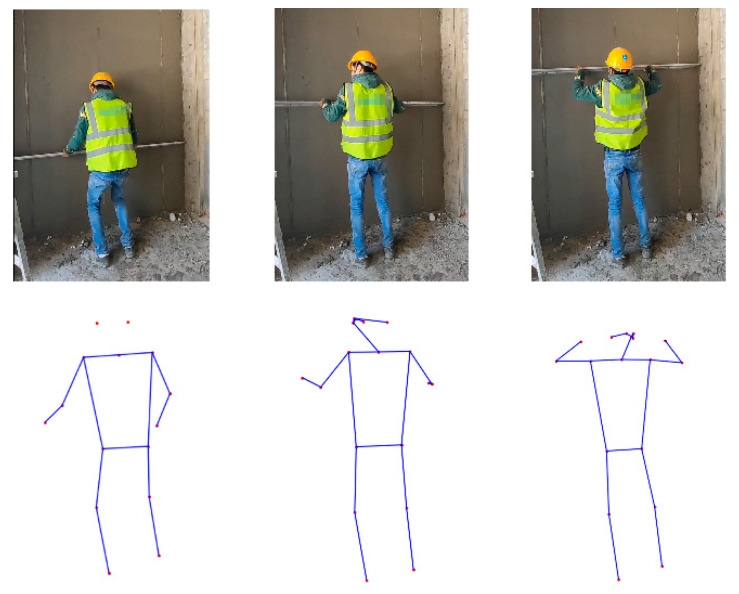
Example of skeletons obtained for an action sequence.

A skeleton graph comprises human joints and joint connectivity, representing human body structures, as illustrated in Figure 2. There are multiple tools to extract skeleton graphs, such MediaPipe, YOLO, OpenPose, etc. Of these, OpenPose, as the first pose estimation method in the deep learning era, has gained widespread usage in various applications. Its simplicity and specific design for pose estimation make it efficient and effective, which allows for real-time skeleton-based human detection [50], particularly in the context of construction sites. OpenPose outputs a skeleton graph as an array with values (X, Y, C) of eighteen joints for two-dimensional (2D) cases. X and Y represent the coordinates on the X-axis and Y-axis of the video frame, respectively, and C is the confidence score of the detected joint. Thus, the estimated joint locations and confidence scores were used as the input, and the raw RGB frames were discarded. Figure 2 shows a graphical explanation of the whole-body joints extracted from OpenPose for 2D cases. Figure 3 illustrates the skeletons corresponding to an action sequence.

### 3.4. ST-GCN-Based Activity Recognition

**Figure 4:**
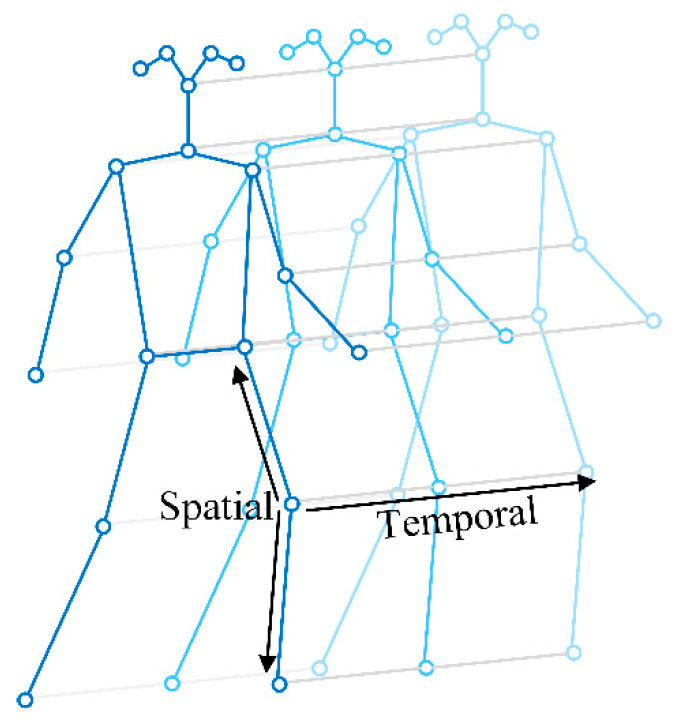
A spatial–temporal graph of a skeleton sequence with intra-skeleton and intra-frame connections, which comprise the spatial and temporal features of the skeleton sequence, respectively.

Spatial–Temporal Graph Convolutional Networks (ST-GCNs) were introduced by Yan et al. [18]. A spatial–temporal graph consists of a sequence of skeletons with connections between the same joint in consecutive frames to track joint movement over time, as shown in Figure 4. Formally, a spatial–temporal graph can be represented mathematically as *G* = (*V*, *E*), which is a graph extending in both the spatial and the temporal dimensions. *V* = {*V*_*ti*_|*t* = 1, …, *T*, *i* = 1, …, *N*} denotes the node set of all the joints in a skeleton sequence, where *T* represents the number of frames and *N* = 18 is the number of joints in each skeleton. The connection set *E* consists of two subsets, including the intra-skeleton connection at each frame and the intra-frame connections. The intra-skeleton connections are denoted as *E*_*S*_ = {(*V*_*ti*_, *V*_*tj*_)∣(*i*, *j*) ∈ *H*}, where *H* is the set of connections that represent human body structures. The intra-frame connections *E_F_* = {(*V*_*ti*_, *V*_(*t*+1) *i*_) ∣*t* = 1, …, *T*, *i* = 1, …, *N*} include all the connections of the same joints in consecutive frames, as shown in Figure 4. The intra-skeleton connections (*E*_*S*_) of each frame and the intra-frame connections (*E_F_*) comprise the spatial and temporal features of the skeleton sequence, respectively.

**Figure 5:**
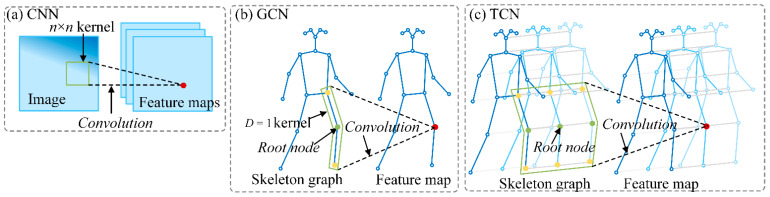
Comparisons between convolution on 2D images and skeleton graphs: (**a**) CNN, (**b**) GCN, and (**c**) TCN. In the case of GCN, the receptive fields of a filter with D = 1 are drawn on the skeleton graph.

**Figure 6:**
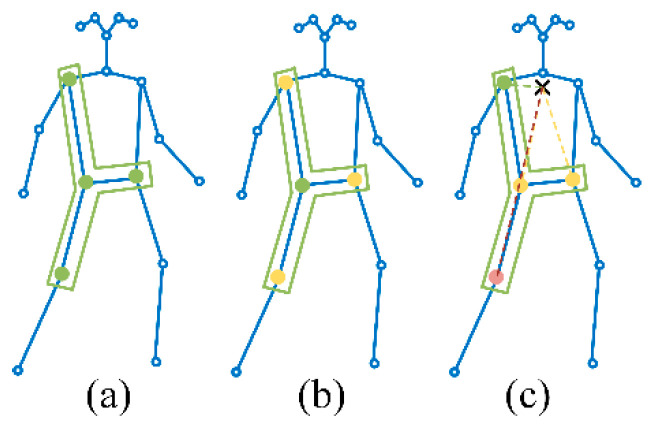
Neighbor set partition strategies: (**a**) uni-labeling, (**b**) distance partitioning, and (**c**) spatial configuration partitioning.

An ST-GCN comprises alternative spatial convolution blocks followed by temporal convolution blocks. Spatial convolution blocks are direct applications of the GCNs, which extend standard image convolution to the cases of graphs. A comparison between convolution on 2D images and on skeleton graphs is vividly shown in Figure 5. On images, the convolution is achieved by adopting a rectangular n×n receptive field, i.e., a so-called kernel, which defines the neighbors of a central location. On graphs, the nodes within a specific distance D compose the neighbor set $B\left(v_{ti}\right)=\{v_{tj}|d\left(v_{tj},v_{ti}\right)\leq D\}$ of a node $v_{ti}$. The concerning node $v_{ti}$ is called the root node. In this work, the neighbor set of a root node is defined as nodes within a distance of one to the root node. The neighbor set is further divided into a fixed number of *K* subsets according to partition strategies, where each subset has a numeric label. Then, the weight function, which provides the weights of each node in the neighbor set for the purpose of computing the convolution, is implemented by mapping nodes in the neighbor set to their subset labels. One of the three typical partition strategies, i.e., uni-labeling, distance partitioning, and spatial configuration partitioning, can be employed according to the modeling capacity and recognition performance, as shown in Figure 6. A more detailed explanation can be found in Ref. [18]. In this study, utilizing the distance portioning strategy, the neighbor set was partitioned into two subsets, one referring to the root node and another comprising the remaining neighbor nodes, as illustrated in Figure 6b. The spatial GCN was extended to the spatial–temporal domain by including temporally connected joints in the neighbor set by $B\left(v_{ti}\right)=\{v_{qj}|d\left(v_{tj},v_{ti}\right)\leq D,\;\left|q-t\right|\leq \Gamma /2\}$, where Γ is the temporal kernel size, which controls the frames to be included in the neighbor set.

## 4. Preliminary Experiments for Activity Recognition 

To validate the effectiveness of the proposed framework, computational experiments exemplified by the plastering work were performed. As a preliminary step, an ST-GCN-based model was trained to recognize different categories of activities in plastering works, which laid the foundation for the action order assessments in Section 5. 

### 4.1. Data Collection and Pre-Process

The plastering work usually consists of the following four activities:

(1) Surface area preparation, including cleaning the wall, removing all dust, and applying an interface agent;
(2) Covering fiberglass meshes to prevent cracks;
(3) Screeding to guide the even application of plaster;
(4) Applying the coat to make the surface uniform and level.

**Figure 7:**
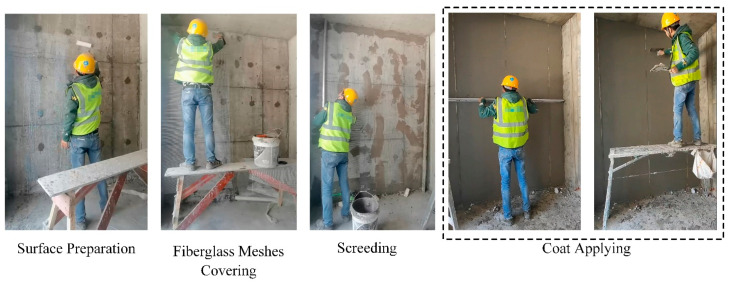
Typical operations of each task in plastering work.

So far, there is no particular video dataset related to plastering works. The dataset in this study was collected from cameras at construction sites. It consisted of videos covering the four primary activities described above, as exemplified in Figure 7. Specifically, in each collection setup, the angle, height, and distance at which the camera was placed were not fixed, since, in real life, the camera angle and distance are not fixed. Moreover, the positions of the workers were not static during the plastering work process, which led to various positions of workers within the frame. Hence, the collected videos included a variety of perspectives. Moreover, the collected video covered multiple workers to exclude the influence of personal operating habits. After the videos were collected, the data pre-processing procedures stated in Section 3.2 were employed to prepare the dataset. Finally, we collected 89 valid videos in total. The numbers of videos for each category of activities and duration range are shown in Table 1. 

### 4.2. Feature Extraction

**Figure 8:**
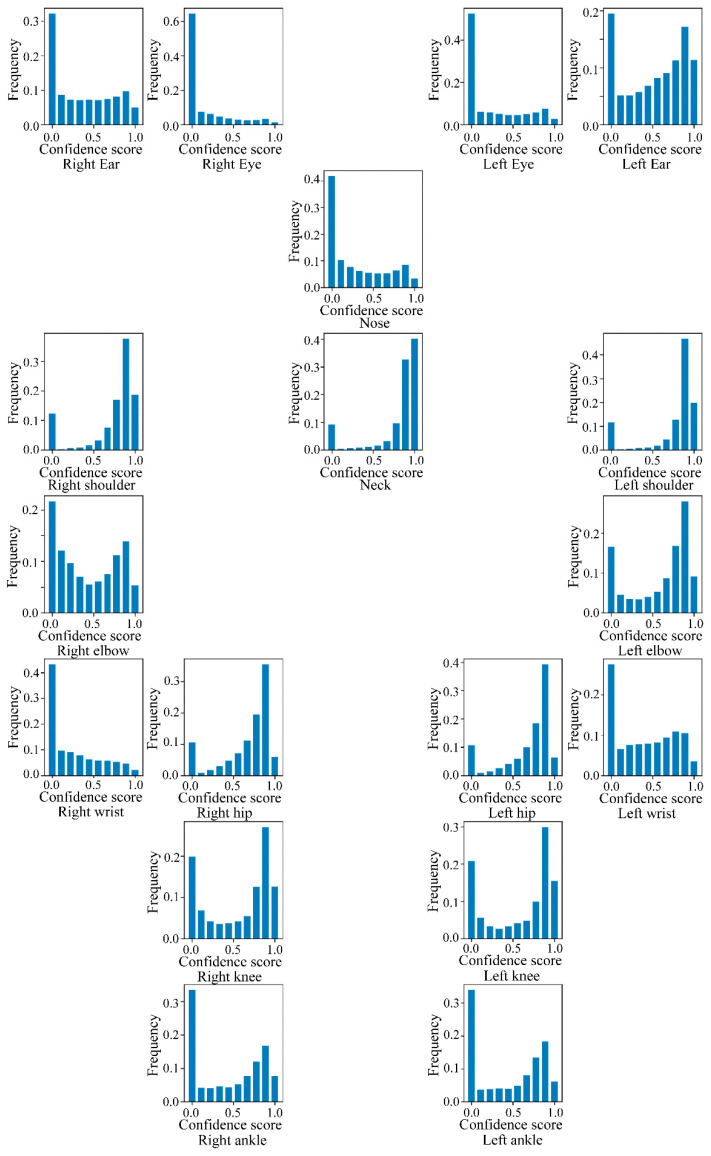
Statistical distribution of confidence scores for 18 human joints.

As stated in Section 3.3, the publicly available OpenPose toolbox was employed in this study to extract 2D coordinates (X, Y) and confidence scores (C) of 18 human joints in every video frame. Then, each joint was represented with a tuple of (X, Y, C), and a skeleton was recorded as an array of 18 tuples. Figure 8 vividly displays the histogram of confidence scores of each joint extracted from the dataset, in which the result of each joint is plotted at the corresponding position in the skeleton. It can be seen that the algorithm can successfully track most of the joints in the main body, like the left and right shoulders, hips, and knees, while the track effect for the left and right wrist is comparatively poor. It is noteworthy that, to be consistent with the reality that some joint recognition results of the surveillance videos can be invalid, the video frames in which the joint features were extracted with low confidence scores were kept. 

### 4.3. Model Training Details

**Figure 9:**
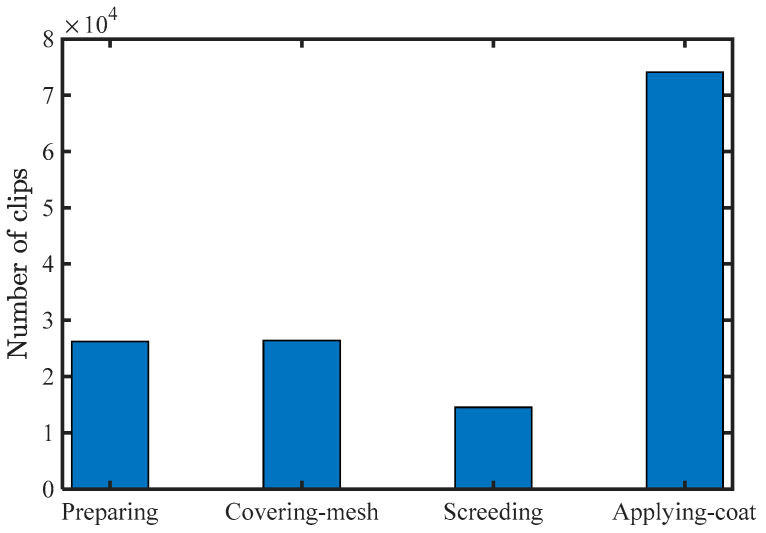
Summary of clips for each activity.

A huge number of videos can efficiently prevent overfitting. However, collecting and annotating a huge dataset is time-consuming and labor-intensive. The dataset was extended to fully utilize the collected videos by extracting different subsequences from a sequence using the sliding window method. Each subsequence composed a video clip, intended to be long enough to ensure the activity was recognizable. In this study, a window of *T* = 128 was used, moving ahead one frame each time. The feature extraction is summarized in Figure 9. The dataset consisted of 26,243 clips for surface preparation, 26,411 clips for mesh covering, 14,532 clips for screeding, and 74,141 clips for coat application. 

In this study, we employed L2-norm as the loss function, since it was preferred in most of the activity recognition and classification cases. The objective of the model training was to minimize the loss function by calculating the gradient with respect to the model parameter. In this study, Adam was used as the optimizer for the loss function, which is a popular algorithm because of its widespread usage and effectiveness in various deep learning tasks [51]. To assess the performance of the AI models during the training process, the dataset was split randomly into two subsets according to fivefold cross-validation: 80% of the video clips for training and 20% for validation.

The training process was deployed on a cloud CentOS system with a memory of 36 GB and an NVIDIA Tesla V100-SXM2 GPU with a memory of 32 GB. The critical code was programmed by Python 3 and its popular deep learning library, named Pytorch. Prior to training, the hyper-parameter initial learning rate was determined to be $10^{-5}$ for mini-batch learning. 

### 4.4. Network Architecture 

**Figure 10:**
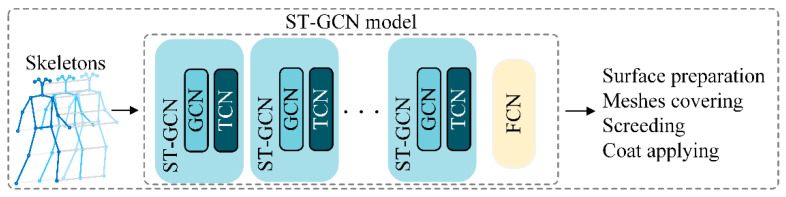
Architecture of spatial–temporal convolutional networks (ST-GCN).

The ST-GCN shown in Figure 10 comprised ten layers of ST-GCN units. The first four layers had 64 output channels, the second three layers had 128 output channels each, and the last three layers had 256 output channels. The convolutional kernel size was set to one. Two techniques were used to avoid overfitting: the residual network mechanism and a dropout layer with a 0.5 drop rate. The input data were represented as a tensor of (*N*, *C*, *T*, *V*, *M*) dimensions, where *N* = 128 was the batch size, *C* = 3 was the joint data (X, Y, C), *T* = 128 was the number of frames in one video clip, *V* = 18 was the number of skeletal joints for each subject, and *M* represented the number of subjects in one clip (here, *M* = 1). Next, the data were fed into the spatial convolutional layer to extract the spatial features of each frame. The spatial convolutional output was then fed into a temporal convolutional layer to extract the temporal features of the same joint throughout successive frames. After the stacks of ST-GCN blocks, the resulting features were fed into a fully connected layer. The model’s output was a vector representing the probability that a given input belonged to a specific class. 

### 4.5. Performance Metrics and Evaluation 

As the ST-GCN model serves as an activity classifier, four metrics were adopted to evaluate its performance, including Accuracy, Precision, Recall, and F1-score, which are typical metrics for classification problems. In a classification task, the class prediction of an item can be either True-Positive (TP), False-Positive (FP), True-Negative (TN), or False-Negative (FN). TP includes items correctly labeled as belonging to the class by the model, while FP includes those incorrectly labeled as belonging to the class. TN consists of items correctly labeled as not belonging to the class, while FN represents items incorrectly labeled as not belonging to the class by the model. Accuracy is the proportion of correct predictions, including TP and TN, among the total items examined.

(1) $$\mathrm{Accuracy}=\frac{\mathrm{TP}+\mathrm{TN}}{\mathrm{TP}+\mathrm{FP}+\mathrm{TN}+\mathrm{FN}}$$

Precision is the number of TP divided by the total number of elements labeled as belonging to the class, i.e., the sum of TP and FP, as

(2) $$\mathrm{Precision}=\frac{\mathrm{TP}}{\mathrm{TP}+\mathrm{FP}}$$

Recall is defined as the number of TP divided by the total number of elements that actually belong to the class, i.e., the sum of TP and FN, as

(3) $$\mathrm{Recall}=\frac{\mathrm{TP}}{\mathrm{TP}+\mathrm{FN}}$$

F1-score is a function of Precision and Recall, as

(4) $$F_{1}=\frac{2}{\frac{1}{\mathrm{Precision}}+\frac{1}{\mathrm{Recall}}}$$

**Figure 11:**
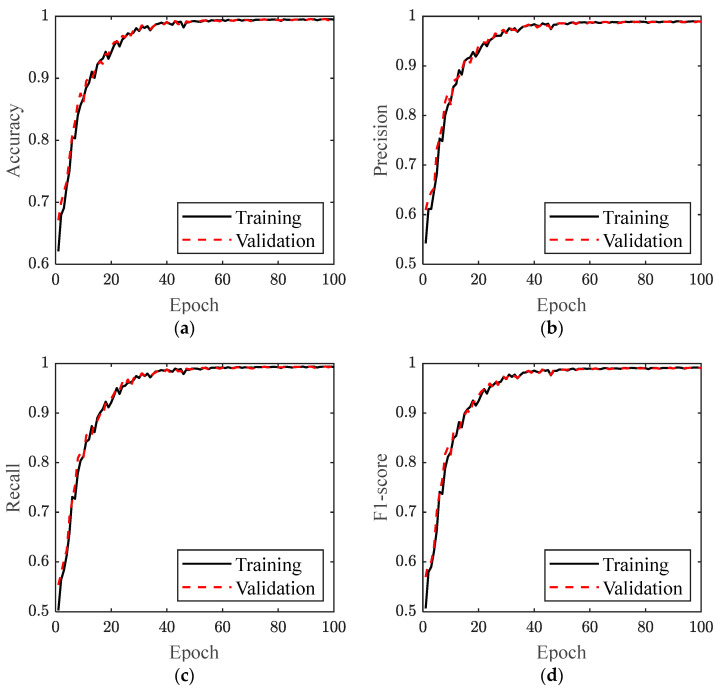
Performance metrics over model training epochs: (**a**) accuracy, (**b**) precision, (**c**) recall, and (**d**) F1-score.

The evolution of the four performance metrics over model training epochs is shown in Figure 11. It can be noticed that the model reached stable results. The results of training and validation accuracy are listed in Table 2. In particular, the Accuracy, Precision, Recall, and F1-score of the validation set were 0.9948, 0.9890, 0.9931, and 0.9909, respectively, which reveals that the trained model was able to accurately recognize different categories of activities in the plastering work.

## 5. Experiments for Activity Order Assessment 

Three independent video clips were used to validate the effectiveness of the constructed ST-GCN model in activity recognition and to illustrate its application in activity order assessment. The three videos represented a process with four activities in the correct order, a process without the activity of mesh covering, and a process in the wrong order, respectively. The three videos consisted of 6540 frames, 4723 frames, and 6540 frames, respectively. 

**Figure 12:**
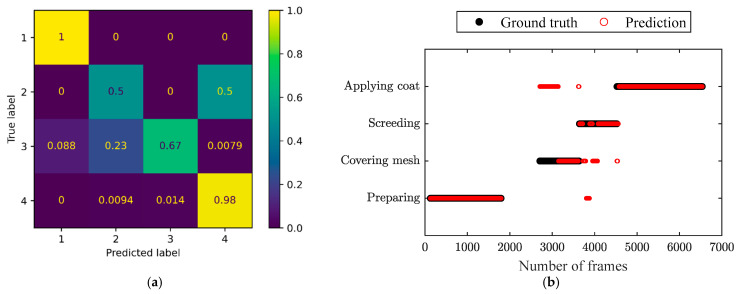
(**a**) Confusion matrix and (**b**) comparison of the prediction and ground truth throughout the frames for the independent testing video clips, representing a process with four activities in the right order. The labels ‘1’ to ‘4’ in the confusion matrix represent ‘Preparing’, ‘Covering mesh’,’ Screeding’, and ‘Applying coat’, respectively.

**Figure 13:**
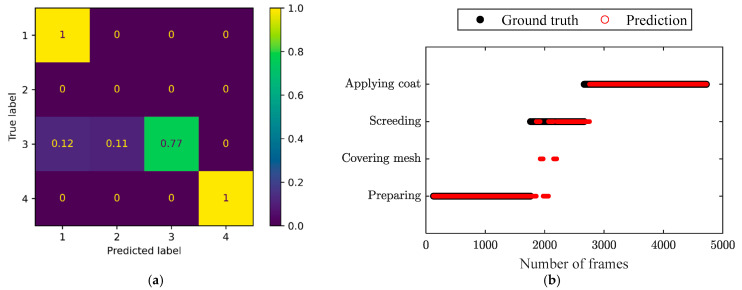
(**a**) Confusion matrix and (**b**) comparison of the prediction and ground truth for the testing video clips, representing a process without covering mesh. The labels ‘1’ to ‘4’ in the confusion matrix represent ‘Preparing’, ‘Covering mesh’,’ Screeding’, and ‘Applying coat’, respectively.

**Figure 14:**
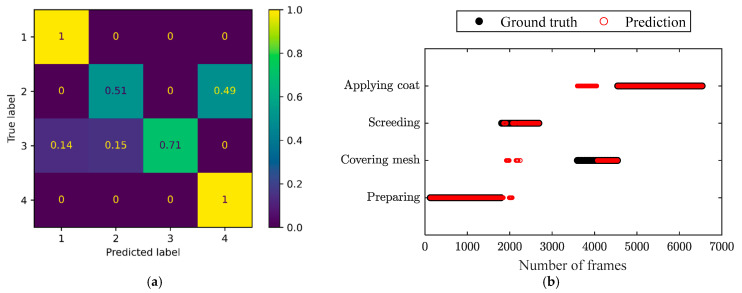
(**a**) Confusion matrix and (**b**) comparison of the prediction and ground truth for the testing video clips, representing a process in which the order of covering mesh and screeding was wrong. The labels ‘1’ to ‘4’ in the confusion matrix represent ‘Preparing’, ‘Covering mesh’,’ Screeding’, and ‘Applying coat’, respectively.

The confusion matrices of the three independent testing video clips and the corresponding comparison of the prediction by the ST-GCN model and ground truth throughout the frames are shown in Figure 12, Figure 13 and Figure 14, respectively. It can be observed in the confusion matrix for each testing video that the activities of surface preparation and coat application were detected with very high accuracy (over 98%). The activity of screeding was recognized with satisfying accuracy ranging from 67% to 77%. The accuracy in identifying mesh covering was relatively low, but acceptable. Moreover, it can be seen that the recognition accuracy for mesh covering was good enough, since the processes with (Figure 12b and Figure 14b) and without mesh covering (Figure 13b) obviously showed distinguished characteristics. Hence, it was easy to judge whether the covering mesh activity was missing. Even though the constructed model might have recognized mesh covering as coat application, the actual corresponding activity order can be identified clearly from Figure 12b and Figure 14b, respectively, which proves the feasibility of this application in activity order assessment.

## 6. Discussion and Conclusions

### 6.1. Performance of Skeleton-Based Activity Order Assessment

The experiment in Section 5 has validated the feasibility of the proposed framework for activity order assessment. The constructed ST-GCN model can efficiently recognize the four categories of activities, even though the model might recognize mesh covering as coat application sometimes. In fact, in the plastering work, before covering the mesh, in order to enhance the cohesion between the fiberglass mesh and the wall, the worker needs to cover the base coat before and after covering the fiberglass mesh. And the operation of covering the base coat is almost the same as that of coat covering. Hence, it is reasonable that the model sometimes recognizes mesh covering as coat application. This deficiency can be improved by a finer activity partition of the whole process. However, the activity order can still be easily identified from the presented results. Thus, the proposed framework can contribute to the realization of contactless, cost-effective, and real-time progress-based quality control in the construction industry.

Furthermore, it was found that the prediction accuracy of the proposed method on test videos was lower than that on training and validation datasets. One of the likely reasons for this fact was that the training dataset and validation datasets were too similar to each other. Once the trained model had been tested on samples from different distributions, the accuracy might have decreased due to the issue of model generalization. This decline in prediction accuracy could be alleviated by selecting a considerable validation dataset, increasing the diversity of the training data, and employing advanced hyper-parameter optimization methods, which could be an important research direction in our future research. 

### 6.2. Potential Applications

(1) Process-based quality control

Conventionally, quality assurance personnel routinely conduct site inspections to ensure that the process and quality comply with regulatory requirements, which is time-consuming and labor-intensive. The proposed framework can be used to implement process-based control. In particular, for concealed work, the construction process can be automatically extracted and assessed from video data, as illustrated by the application in the plastering works in this study. Moreover, state information, including activity durations and transitions, can be further extracted to evaluate whether an operation follows its method statement [41]. For example, in compacting concrete, the cycle duration of insertion and withdrawal can be withdrawn from the model to assess whether the vibration is sufficient.

(2) E-learning in construction training

Labor productivity highly depends on the levels of skills and abilities of the laborers. A labor training program has been developed with the scope of improving the productivity and performance of the labor force in construction, which consists of the following processes [52]:

(1) Designing labor training exercises and training delivery methods;
(2) Measuring the relative weights of labor training exercises;
(3) Assessing labor competencies;
(4) Developing a performance score system and grading scheme for laborers;
(5) Training reinforcement.

The activity recognition and activity order assessment framework proposed in this paper can be a functional tool and is expected to facilitate the assessment of labor competencies by automatically detecting the competencies of planned construction sequences.

### 6.3. Limitations and Future Directions

Since the proposed approach is an early attempt at process-based quality control via ST-GCN in the construction industry, several limitations call for further investigation. Due to external factors, such as the distance and angle of the camera, the people in the surveillance video will be obscured, and sometimes occluded [53]. A solution to this limitation is the combination of vision-based techniques with sensor-based technologies. The application in a multi-person environment is another critical factor. Exploring an efficient recognition and tracking strategy for multiple people deserves further investigation.

### 6.4. Conclusions

This study introduced a novel approach for leveraging surveillance cameras to automatically enact process-based quality control of concealed construction works using extracted skeleton data and the ST-GCN algorithm to recognize different categories of activities. Experiments were conducted using field videos of plastering works to verify the effectiveness of the proposed method. An ST-GCN model was first trained to recognize the four primary activities in plastering works. The model achieved 99.48% accuracy on the validation set, which indicates that the model can attain relatively high accuracy in terms of recognizing activities in plastering works. Then, extra experiments were designed to illustrate that the order of activities can be assessed based on the activity-recognizing results. These results indicate that the proposed CV-based activity recognition framework could be a valuable tool in automatic quality control, and may contribute to more convenient and effective quality management of concealed work.

## Figures and Tables

**Table 1:** Summary of collected videos for ST-GCN model construction.

Activity	Number of Videos	Duration in Total (s)	Maximum Duration (s)	Minimum Duration (s)
Surface preparation	20	1189	122	7
Fiberglass mesh covering	12	1151	269	14
Screeding	29	725	182	7
Coat applying	28	3185	442	9

**Table 2:** Performance metrics of the ST-GCN model on the training and validation sets.

Index	Accuracy	Precision	Recall	F1-Score
Training	0.9947	0.9890	0.9930	0.9909
Validation	0.9948	0.9890	0.9931	0.9909

## Data Availability

Some or all data, models, or code that support the findings of this study are available from the corresponding author upon reasonable request.
